# Characterization of slow-cycling cells in the mouse cochlear lateral wall

**DOI:** 10.1371/journal.pone.0179293

**Published:** 2017-06-20

**Authors:** Yang Li, Kotaro Watanabe, Masato Fujioka, Kaoru Ogawa

**Affiliations:** 1 Department of Otorhinolaryngology, School of Medicine, Keio University,35 Shinanomachi, Shinjuku-ku, Tokyo, Japan; 2 Department of Otolaryngology-Head and Neck Surgery, the Second Affiliated Hospital, Xi'an Jiaotong University, Xi Wu Lu, Xi'an, China; Texas A&M University, UNITED STATES

## Abstract

Cochlear spiral ligament fibrocytes (SLFs) play essential roles in the physiology of hearing including ion recycling and the generation of endocochlear potential. In adult animals, SLFs can repopulate after damages, yet little is known about the characteristics of proliferating cells that support SLFs’ self-renewal. Here we report in detail about the characteristics of cycling cells in the spiral ligament (SL). Fifteen P6 mice and six noise-exposed P28 mice were injected with 5-bromo-2′-deoxyuridine (BrdU) for 7 days and we chased BrdU retaining cells for as long as 60 days. Immunohistochemistry revealed that the BrdU positive IB4 (an endotherial marker) negative cells expressed an early SLF marker Pou3f4 but negative for cleaved-Caspase 3. Marker studies revealed that type 3 SLFs displayed significantly higher percentage of BrdU^+^ cells compared to other subtypes. Notably, the cells retained BrdU until P72, demonstrating they were dividing slowly.

In the noise-damaged mice, in contrast to the loss of the other types, the number of type 3 SLFs did not altered and the BrdU incorporating- phosphorylated Histone H3 positive type 3 cells were increased from day 1 to 14 after noise exposure. Furthermore, the cells repopulating type 1 area, where the cells diminished profoundly after damage, were positive for the type 3 SLF markers. Collectively, in the latral wall of the cochlea, type 3 SLFs have the stem cell capacity and may contribute to the endogenous regeneration of lateral wall spiral ligament. Manipulating type 3 cells may be employed for potential regenerative therapies.

## Introduction

Mammalian cochlear spiral ligament (SL) fibrocytes (SLFs) of the mesenchymal non-sensory regions play essential roles in the cochleae physiology of hearing, including the transport of potassium ions to generate an endocochlear potential in the endolymph [1–3]. Recent studies indicate that SLFs are involved in acute and permanent sensorineural hearing loss [4, 5]. In mouse and gebil models of age-related hearing loss, loss of SLFs proceeds the degeneration of other cell-types within the cochlea [6, 7]. DFN3, an X chromosome-linked nonsyndromic mixed deafness, exhibits severe ultrastructural alterations in SLFs, which results in a dramatic reduction in endocochlear potential and a profound hearing loss [8, 9].

Accumulating evidence suggests that the cochlear fibrocytes may have regenerative capacity, which is a potential therapeutic target for the deafness associated with lateral wall degeneration. Under normal conditions, cochlear fibrocytes can divide continuously, although the proliferation capacity decreases when the animal is at an advanced age [10]. However, after damage, the proliferation capacity increases even in adult animals [4, 5, 10, 11]. A recent study also proved the increased proliferation of SLFs, particularly of type 2 fibrocytes, the damage of which is severest in the model, by an experiment with 5-bromo-2-deoxyuridine (BrdU) after 3-nitropropionic acid (3-NP) damage. Delayed spontaneous regeneration and partial hearing recovery are accompanied by morphological remodeling of the cochlear lateral wall [12]. Studies have shown that the lateral wall may harbor multi-potent cells [13, 14]. Those *in vitro* studies have proved the existence of stem cells that turn into several cochlear cell types which may ultimately support the recovery of structure and function as shown in several *in vivo* reports [4, 10, 11, 13, 14]. However, little is known about the characteristics of the spatiotemporal distribution of cochlear lateral wall stem cells.

On the other hand, there is quite a number of evidence that resident populations of relatively undifferentiated adult stem cells play important roles in maintaining many highly regenerative tissues [15, 16]. The key features of stem cells are multipotency, slow cycling and self-renewal. In the absence of a specific cell marker, identifying stem cells by their slow-cycling property has been recognized as a new approach to detect tissue-specific stem cells in various organs. With this approach, BrdU, a synthetic thymidine analogue has been used as DNA labeling that incorporates into newly synthesized DNA during the S phase of mitosis, and dilutes out over time during cell division. After a long labelling and chasing period, rapidly dividing cells lose the label and only infrequently dividing cells take up the label and retain it by cycling slowly or becoming quiescent. This technique provides a powerful way to detect stem cells in many organs, including corneal epithelia stem cells, hair follicular epithelial stem cells, hepatic stem cells, mammary epithelial stem cells, and cochlear tympanic border cells and vocal ford stem cells [17–23].

In the present study, we ask if the proliferating cells in the cochlear lateral wall have the characteristics of stem cells and which cell types in the lateral wall are the stem cells. Along with the long-term BrdU pulse-chase labelling, we performed immunostaining for specific markers of SLFs to identify the spatiotemporal distributions of label-retaining cells in naïve and injured mouse cochlear fibrocytes. The results reveal that the slow cycling cells, or potential stem cells, can be seen most frequently in the type 3 fibrocytes. We also exhibit the evidence that the number of BrdU incorporating- phosphorylated Histone H3 (PH3) positive cells increases in type 3 area at day 14 after noise exposure, and the BrdU^+^IB4^-^ type 3 SLFs repopulate type 1 area where fibrocytes decreased profoundly when exposed to intense noise. Collectively, we conclude that the type 3 cells of the spiral ligament fibrocytes contain stem cell population in the cochlear lateral wall that may be employed for potential regenerative therapies.

## Materials and methods

### Animals

All mice were purchased from Japan SLC (Shizuoka, Japan) and maintained in the animal care facility of Keio University. Twenty-one male postnatal six-day-old C57BL/6J mice and eleven male four-week-old C57BL/6J mice were used in the study. All animal experiments were approved by the ethics committee of Keio University Union on Laboratory Animal Medicine in accordance with the Guide for the Care and Use of Laboratory Animals (National Institute of Health, Bethesda, MD).

### BrdU labeling

Mice were injected intraperitoneally with 50 mg/kg BrdU (Sigma-Aldrich) at 12 hour intervals for 7 consecutive days. To detect the slow cycling cells in a long chasing time of BrdU, fifteen P6 mice were sacrificed, and their cochleae were harvested at 1 day, 2 weeks, 4 weeks, 8weeks after injection (n = 3 per time points). To detect the slow cycling cells after acoustic trauma, six P28 mice were injected intraperitoneally with BrdU at two days after noise-exposure, sacrificed and dissected at 1 day and 2 weeks after injections (n = 3 per time points). At the time of sacrifice, mice were deeply anesthetized and transcardially perfused with 0.01 M phosphate buffer saline (PBS, pH 7.4), followed by fixative solution of 4% paraformaldehyde (PFA, pH 7.4).

### Noise exposure

Animals were exposed for 2 hr to a 120 dB SPL of octave band noise centered at 4 kHz in a ventilated sound-exposure chamber. The sound chamber was fitted with a speaker (HFD-260-8, TOA) that was driven by a noise generator (SF-08, RION) and a power amplifier (DUAL POWER AMPILFIER, P-120D, TOA and RDQ-2031, Roland RAD). Sound levels were calibrated at multiple locations within the sound chamber to ensure uniformity of the stimulus (type 2203 precision sound level meter, type 4134 microphone; Bruel and Kjaer Instruments) and analyzed by a fast Fourier transform network analyzer with a linear scale. The stimulus intensity varied by≤3 dB across the measured sites within the exposure chamber. During noise exposure, noise levels were monitored with a sound level meter, a pre-amplifier, and a condenser and a microphone (NL-42, RION). The microphone was positioned within the cage approximately at the level of the animal’s head.

### Auditory brainstem response (ABR) recordings

ABR recordings were performed with an extracellular amplifier Digital Bioamp system (BAL-1; Tucker-Davis Technologies, Alachua, FL), and wave form storing and stimulus control were performed with Power-Lab systems Scope software (PowerLab 2/20; AD Instruments, Castle Hill, Australia). Sound stimuli were produced with a coupler type speaker (ES1spc; Bioresearch Center, Nagoya, Japan), which was inserted into the external auditory canal of each mouse. Mice were anesthetized with ketamine (80 mg/kg, i.p.) and xylazine (15 mg/kg, i.p.), and then implanted with stainless steel needle electrodes, which were placed at the vertex and ventrolateral to the left and right ears. Sound stimuli consisted of 15-m sec tone bursts with a rise-fall time of 1 m sec at frequencies of 4, 8, 12 or 20 kHz. Generally, the ABR waveforms were recorded for 12.8 ms at a sampling rate of 40,000 Hz using 50–5,000Hz band-pass filter settings; waveforms from 1,024 stimuli at a frequency of 9 Hz were averaged. The ABR waveforms were recorded at 5 dB SPL intervals. Threshold was defined as the lowest stimulation intensity that yielded a repeatable waveform based on an identifiable ABR wave III or IV, whichever demonstrated greater sensitivity. Each ear was tested separately, and thresholds were averaged across the ears for each frequency in each animal.

### Immunohistochemistry

The cochleae were fixed with 4% PFA at 4°C overnight followed by decalcified in 0.5 M EDTA (pH 7.5) at room temperature for 7 days. Then the specimens were dehydrated in a graded ethanol series, embedded in OCT compound (SAKURA, Finetek Japan, Tokyo). Seven-micrometer sections were obtained with a cryostat. All sections were heated in 10μM citrate buffer (pH 6.0) for 15 minutes for antigen retrieval, then permeabilized in 0.03% triton-X for 1 hour and blocked in 10% donkey or goat normal serum for 1 hour at room temperature, followed by incubation with primary antibodies in TBST for overnight at 4°C. Primary antibodies used were as follows: anti-BrdU (1:100, Abcam), anti-IB4 (1:100, Vector Laboratories), anti-S100β (1:200, Neo Markers), Aqp1 (1:300, Santa Cruz), anti-Na,K-ATPase α1 (1:500, Novus), anti-Cx26 (1:300, Zymed), anti-Ki67 (1:200, Abcam), anti-Pou3f4 (1:200, Atlas Antibody), anti-NKCC1(1:200, Abcam) and-phosphorylated Histone H3 (1:100, Abcam). Secondary antibodies used were Alexa 488, 568 or 647 conjugated with anti-rabbit/mouse/rat IgG (Molecular Probes). For counterstaining, 2 mg/mL 4’, 6’-diamidino-2-phenylindole dihydrochloride (DAPI, Sigma-Aldrich) was used. Images were obtained using a fluorescence microscope (E600, Nikon) and a confocal laser scanning microscope (LSM 710, Zeiss). Observations were conducted at the basal turn and the apical side of the middle turn of the cochlea.

### Cell counts and statistical analysis

We prepared three consecutive mid-modiolar sections from each cochleae. For the quantitative analyses, we used two or more slides per cochleae after immunohistochemistry. Multiple comparisons with the Tukey-Kramer method were performed for pair wise analyses. A p-value less than 0.05 were considered statistically significant. All data were obtained using JMP Pro 11.1 (SAS, Cary, NC).

## Results

### 1. Persistence of long-term BrdU label-retaining cells in the lateral wall of cochleae

**Fig 1:**
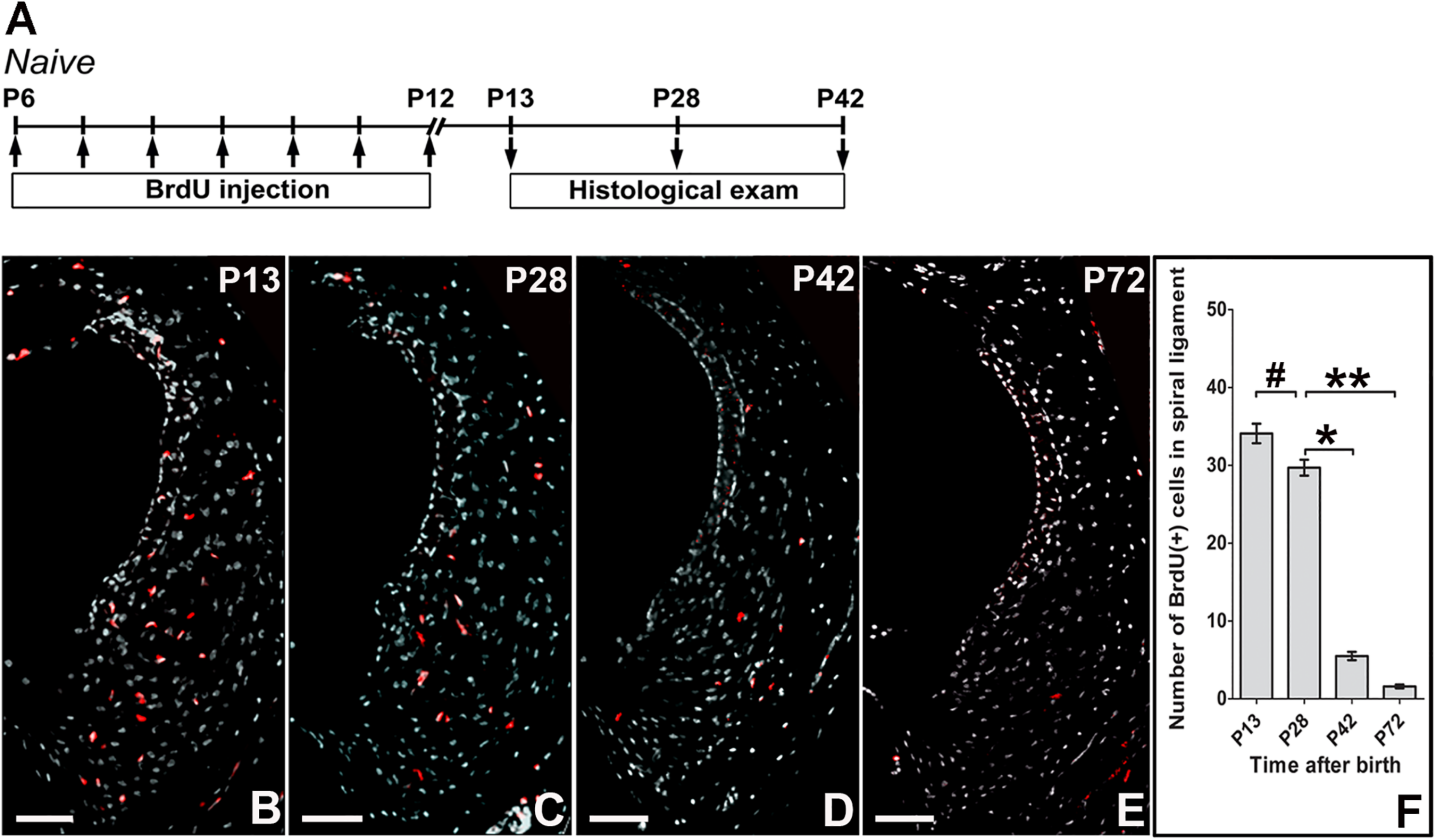
Distribution of BrdU-positive cells in the lateral wall of cochleae. Experimental design indicating the duration of BrdU treatment and observation time points in naive mice (A), BrdU was injected daily in between P6 and 12 and the remained BrdU was examined by immunohisitochemistry. Representative examples of immunohistochemistry for BrdU on P13(B), P28(C), P42(D), P72(E) after BrdU injection; BrdU-positive cells were located in the SL and Stv. Their number was significantly reduced in the SL at P42 and P72 (F). ^#^P > 0.05, **P < 0.01, *P < 0.05. Scar bar = 100μm.

Previous studies have shown that the stem cell capacities of the cochlear lateral wall cells are high in the neonatal stages and decrease dramatically by adulthood [10]. Also, it is reported that the neonatal cochlear fibrocytes can be propagated [10, 24]. We therefore primarily labeled cochlear cells by BrdU in between P6 and P12 and examined where and how long the cells retain BrdU (Fig 1A). At P13 and P28, numerous BrdU^+^ cells were located in SL of cochleae (Fig 1B and 1C). The number of BrdU^+^ cells in SL decreased significantly by P42 and P72. However, even at P72, BrdU^+^ cells were still observed in SL (Fig 1D–1F).

### 2. Characterization of long-term BrdU retaining cells in the lateral wall of cochleae

**Fig 2:**
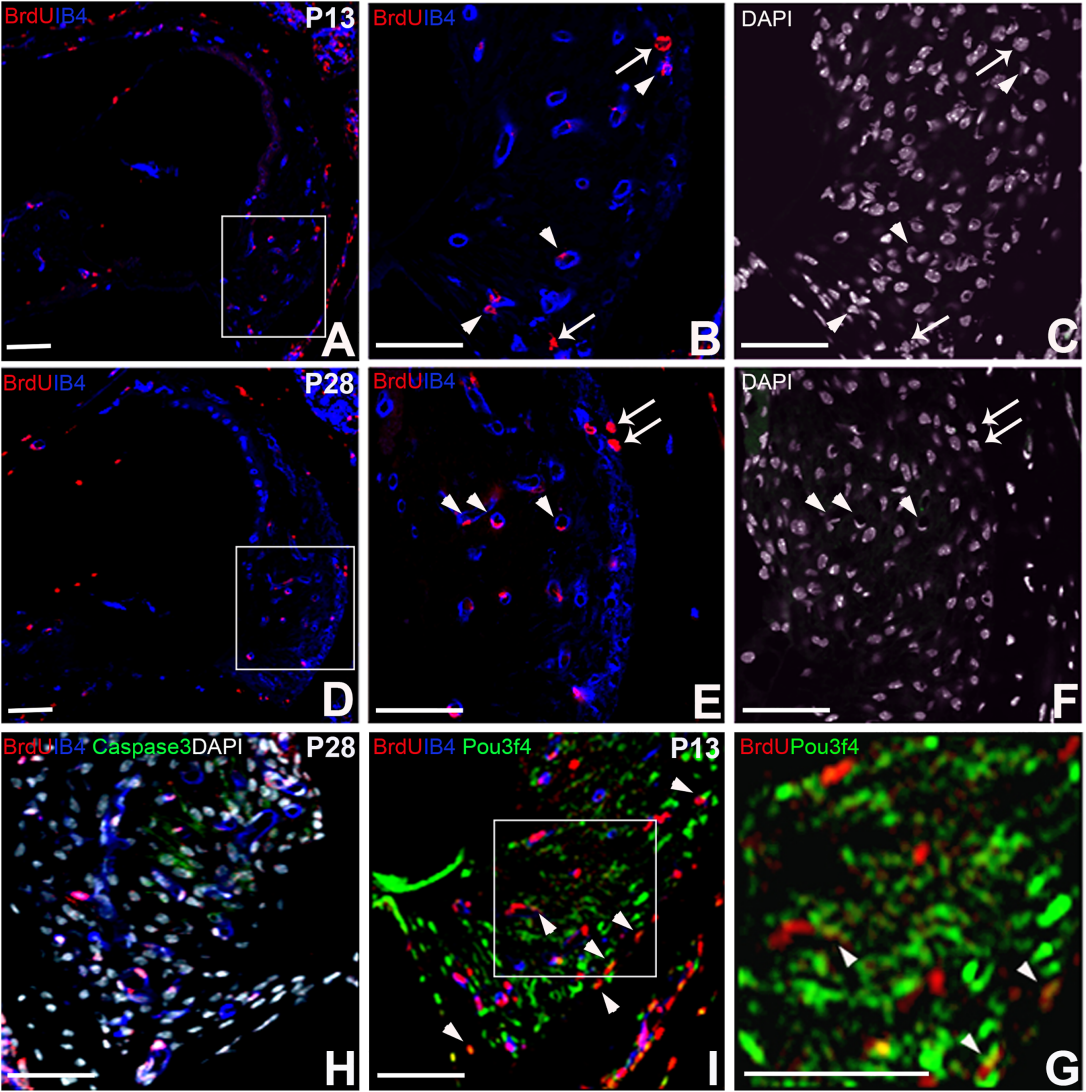
Characterization of long-term BrdU label retaining cells in the SL. Double labelings of BrdU/IB4 (A-F), triple labeling of BrdU/IB4/Caspase3 (H), BrdU/IB4/Pou3f4 (I and G) were shown. Nuclei were stained using DAPI. BrdU positive cells were co-labeled with anti-DAPI (B, C, E, F and H). 11.44 ± 0.80% (at P13) or 9.33 ± 1.03% (at P28) of BrdU^+^ cells were also positive for IB4^+^ (B, E, arrowheads). BrdU^+^IB4^-^ cells were negative for Caspase3, indicating that the labeling was not due to apoptosis. 3.52 ± 0.68% (at P13) of BrdU^+^IB4^-^ cells were positive for Pou3f4 (I and G arrowheads), indicating the SL fibrocytes proliferate during the neonatal stages. Scar bar = 100μm.

We next characterized BrdU retaining cells by co-labelling with several different markers. First, we labeled with IB4, a specific marker of vascular endothelial cells (Fig 2A–2F). As shown in Fig 2B (P13) and 2E (P28) as pointed by arrows, many of the BrdU labeled cells were positive for anti-IB4 (Fig 2B and 2E, arrowheads), indicating that BrdU^+^cells at the stages contain both endothelial lineages and other cell types.

Pou3f4, the mouse orthologue of the POU3f4 gene, was expressed in the periotic mesenchyme, i.e., cochlear lateral wall cells, Reissner membrane and modiolar bone in the early developing inner ear. At P13, some BrdU^+^IB4^-^ cells were co-labeled with anti-Pou3f4 (Fig 2I and 2G, arrowheads), indicating that these BrdU^+^IB4^-^ cells were immature cochlear SLFs. There were no BrdU^+^IB4^-^ cells co-labeled with anti-Caspase3, a marker of apoptosis, indicating that BrdU^+^IB4^-^cells were not dying cells (Fig 2H).

### 3. Cell types of BrdU retaining cells in the SL fibrocytes

The SL in the lateral wall consists of five subtypes of fibrocytes based on their structural features, immunostaining patterns, and general location (Table 1). We performed immunohistochemistry for S100, Na,K-ATPase α1, Aqp1 and Cx26, to determine fibrocyte types and quantified the percentage of individual types at P13, P28 and P42.

**Table 1:** Immunostaining patterns in spiral ligament fibrocytes of the cochlea.

	Type of fibrocytes				
	Type 1	Type 2	Type 3	Type 4	Type 5
Na,K-ATPase α1	–	+		+	+
Cx26	+				+
S100	+	+		+	+
Aqp1			+		

#### 3.1 Na,K-ATPase α1 and Cx26 immunohistochemistry

**Fig 3:**
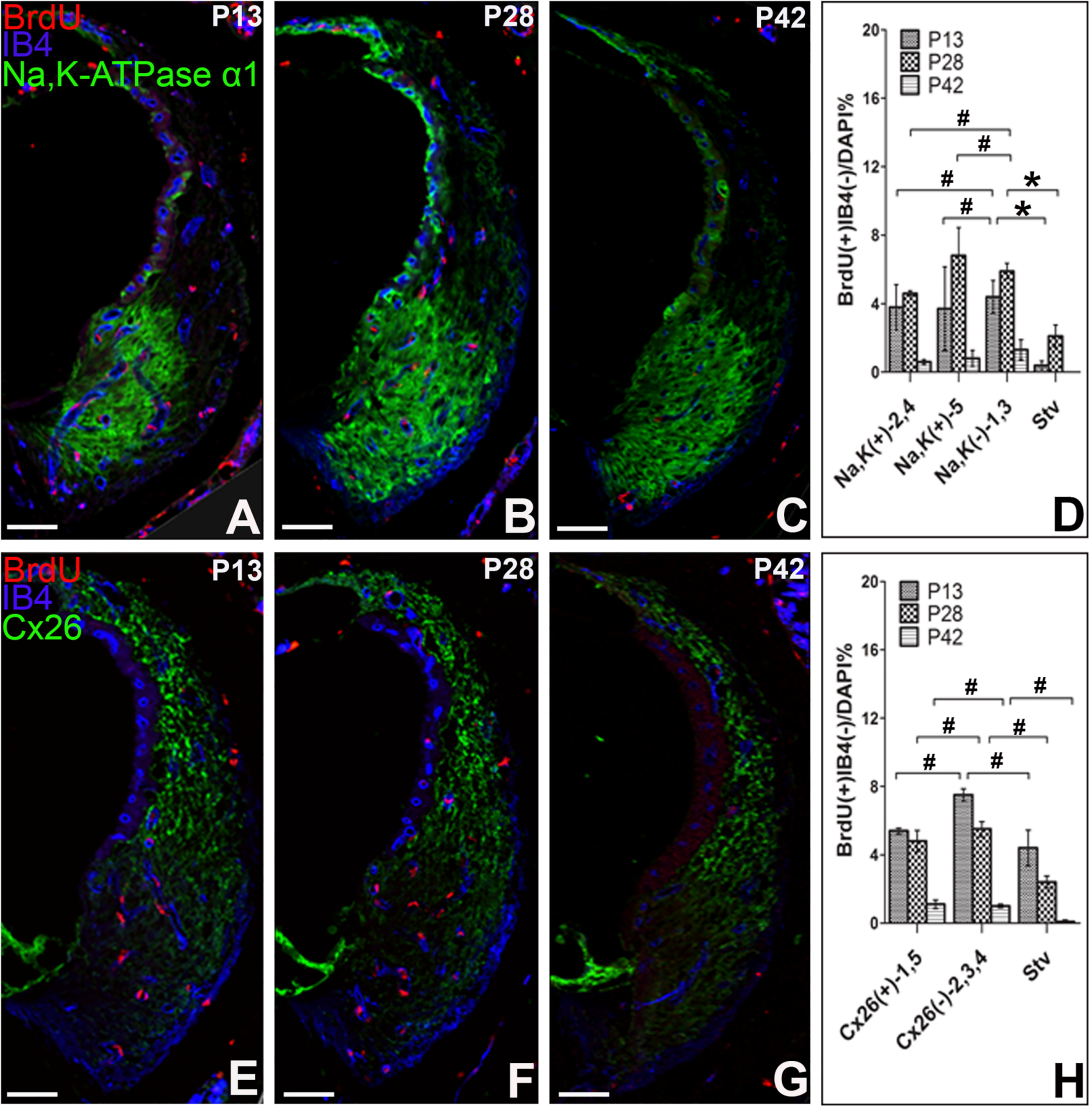
Distribution of BrdU^+^IB4^-^ fibrocytes at Na,K-ATPase α1 or Cx26 positive area. Triple immunostaining for BrdU, IB4, Na,K-ATPase α1 (A-C) or Cx26 (E-F) were performed at P13, P28 and P42 after BrdU administration. The percentage of BrdU^+^IB4 cells was compared among Na,K-ATPase α1 positive area, Na,K-ATPase α1 negative area and Stv, or among Cx26 positive area, Cx26 negative area and Stv. ^#^P>0.05, **P<0.01, *P<0.05. Scar bar = 100μm. (n = 3 per each group).

Using Na,K-ATPase α1 immune staining, the lateral wall of the cochleae was divided into positive area (type 2, 4, 5), negative area (type 1, 3) and Stria vascularis (Stv). Since type 5 area was a discrete area and easy to be distinguished, we further subdivided Na,K-ATPase α1 positive area into type 2, 4 area and type 5 area. At P13, the percentage of BrdU^+^IB4^-^ cells in type 2, 4 area, type 5 area and type 1, 3 area was 3.80% ± 1.31%, 3.70% ± 2.46% and 4.4% ± 0.96% respectively. At P28, the percentage raised slightly to 4.60% ± 0.18%, 6.80% ± 1.65% and 5.90% ± 0.45% respectively, and decreased obviously to 0.60% ± 0.13%, 0.80% ± 0.41% and 1.30% ± 0.59% respectively at P42. However, there were no significant differences in the distribution of BrdU^+^IB4^-　^fibrocytes among these areas at each time point, except Stv area (0.40% ± 0.24% at P13, 2.10% ± 0.65% at P28, 0.00% at P42) (Fig 3A–3D). In addition, the lateral wall of the cochleae was divided into positive area (type 1, 5), negative area (type 2, 3, 4) and Stv by Cx26 staining. At P13 and P28, the distribution of BrdU^+^IB4^-^ cells was 5.40% ± 0.17% and 4.80% ± 0.62% in Cx26 positive area, 7.50% ± 0.35% and 5.50% ± 0.45% in Cx26 negative area, and 4.40% ± 1.04% and 2.40% ± 0.35% in Stv respectively. Although at P42, the percentage decreased obviously to 1.10% ± 0.25% in Cx26 positive area, 1.00% ± 0.10% in Cx26 negative area and 0.10% ± 0.18% in Stv, there were no significant differences in the distribution of BrdU^+^IB4^-^ fibrocytes among these areas at each time point (Fig 3E–3H).

#### 3.2 S100 and Aqp1 immunohistochemistry

**Fig 4:**
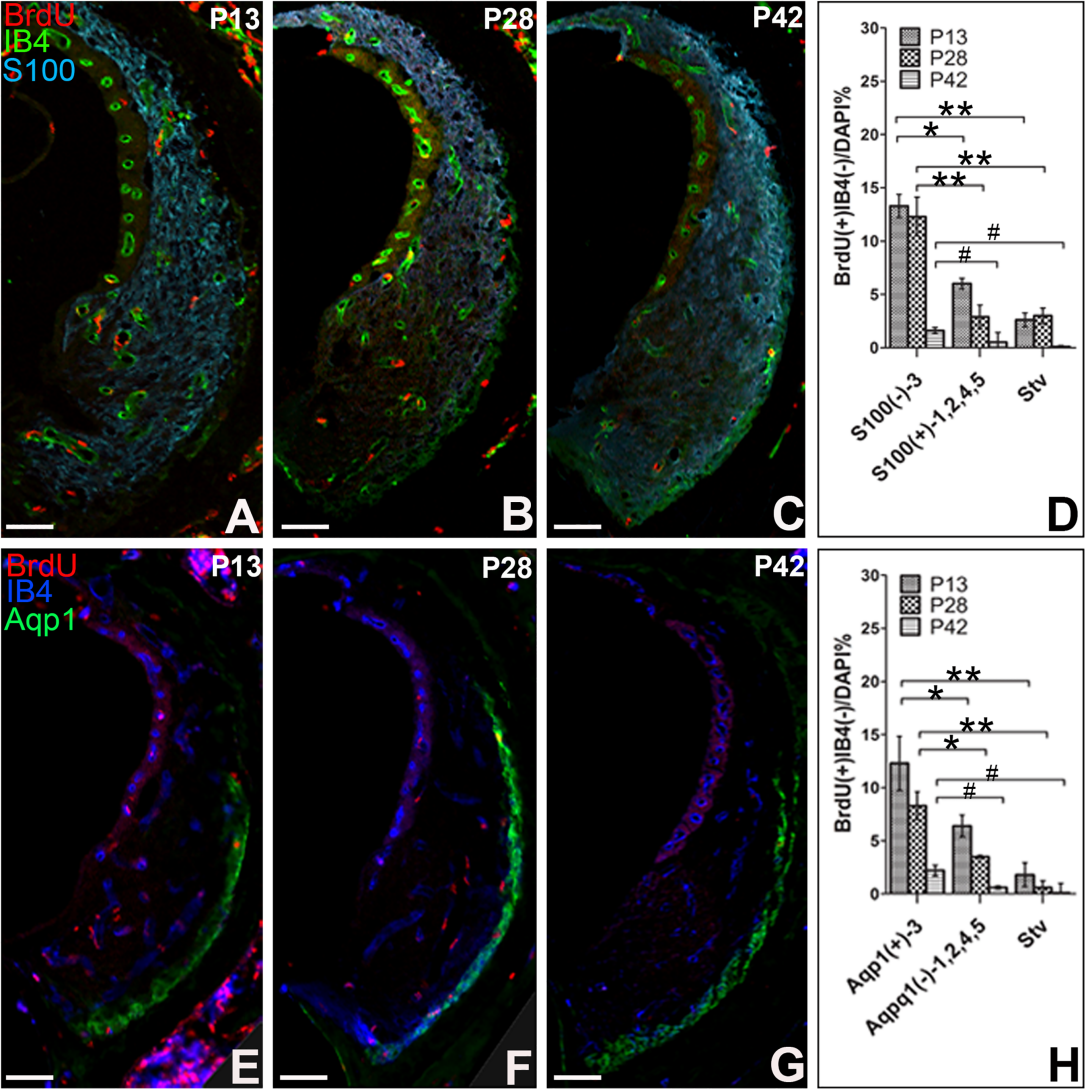
Distribution of BrdU^+^IB4^-^ fibrocytes at S100 or Aqp1 positive area. Triple immunostaining for BrdU, IB4, S100 (A-C) or Aqp1 (E-F) were performed at P13, P28 and P42 after BrdU administration. The percentages of BrdU^+^IB4 cells were compared among S100 positive area, S100 negative area and Stv, or among Aqp1 positive area, Aqp1 negative area and Stv. ^#^P > 0.05, **P < 0.01, *P < 0.05. Scar bar = 100μm.

The lateral wall of the cochleae was divided into S100^+^ area (type 1, 2, 4, 5), S100^-^ area (type 3) and Stv, or Aqp1^+^ area (type3), Aqp1^-^ area (type1, 2, 4, 5) and Stv by S100 or Aqp1 staining. At P13, the percentage of BrdU^+^IB4^-^ cells in S100^–^ area was 13.3% ± 1.08%, which was significantly higher than that in S100^+^ area (6.0% ± 0.51%) and that in Stv (2.6% ± 0.65%). At P28, the S100^-^ area still had higher percentage of BrdU^+^IB4^-^ cells than other two areas. However, at P42, the percentage in S100^-^ area dropped to 2.5% ± 0.67%, and there were no significant differences among these three areas (Fig 4A–4D). Similar results were observed in the triple labeling with Aqp1. At P13 and P28, the percentage of BrdU^+^IB4^-^ cells in Aqp1^+^ area was 12.30% ± 2.54% and 8.30% ± 1.31% respectively, which were significantly higher than that in Aqp1^–^ area (6.40% ± 1.02% at P13, 3.50% ± 0.14% at P28) and that in Stv (1.80 ± 1.12% at P13, 0.60 ± 0.63% at P28). At P42, the percentage in Aqp1^+^ area dropped to 2.20% ± 0.49%, and there were no significant differences among these three areas (Fig 4E–4H), which further demonstrated that compared to other areas, there was higher percentage of distribution of BrdU^+^IB4^-^ cells in type 3 SFLs before P28 time point.

#### 3.3 Spatiotemporal distributions of BrdU-labelled SLfibrocytes

**Fig 5:**
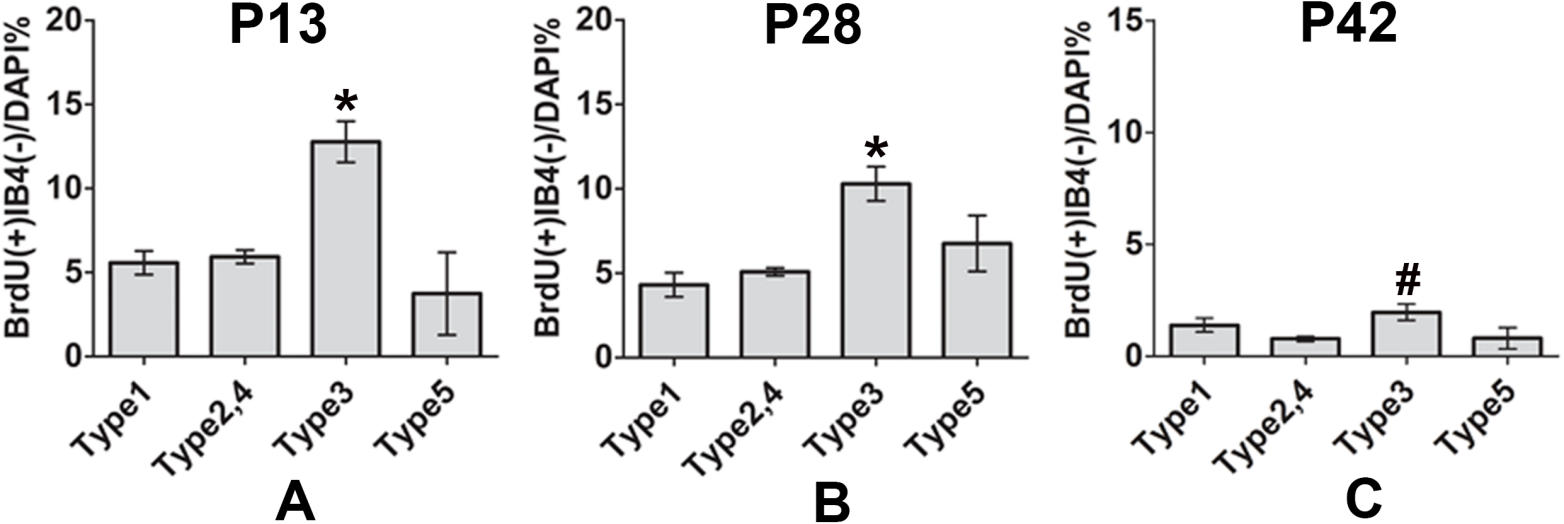
Spatio-temporal changes in the distribution of BrdU^+^ cells. Comparison of the percentages of BrdU^+^IB4^-^ cells in Type 1, Type 2, 4, Type 3, Type 5 region at P13(A), P28(B) and P42 time point. ^#^P > 0.05, *P < 0.05.

Collectively, we quantified the percentage of BrdU^+^IB4^-^ fibrocytes in each types at P13, P28 and P42. We determined the individual types of fibrocytes as follows: Type 1: Cx26^+^S100^+^ cells; Type 2, 4: Na,K-ATPase α1^+^ S100^+^ cells; Type 3: Aqp1^+^IB4^-^ cells; Type 5: Na,K-ATPase α1^+^ Cx26^+^ S100^+^ cells. In the spatial distribution, type 3 SLFs owned higher percentage of BrdU^+^ cells compared to type1, type 2, 4 and type 5 SLFs (Fig 5A–5C).

#### 3.4 Long-term retaining of BrdU in type 3 SLFs

**Fig 6:**
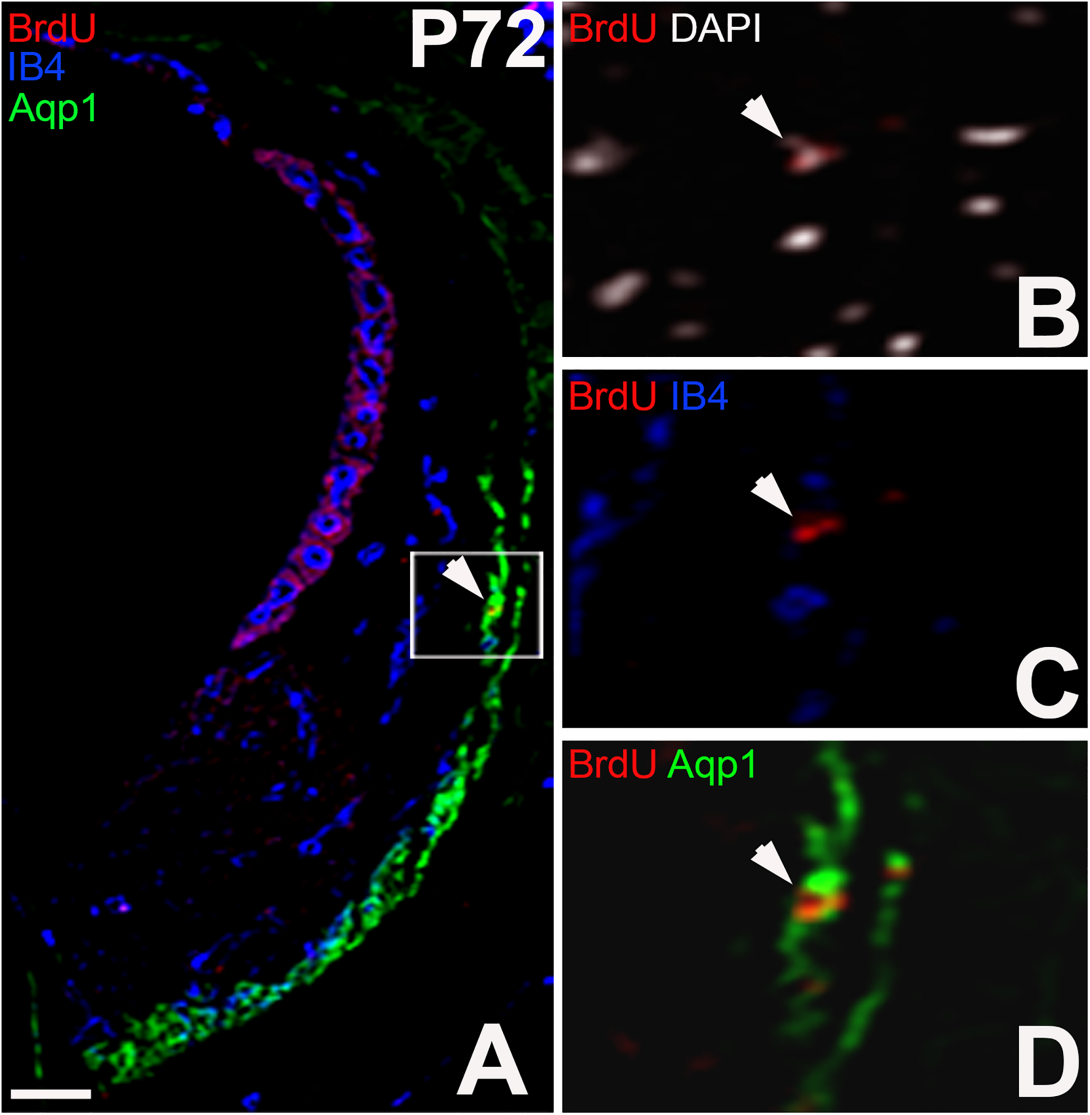
Retained expression of BrdU^+^IB4^-^ cells in type 3 area at P72. (A) Triple immunostaining for BrdU, IB4 and Aqp1. (B, C, D) Enlarged image of corresponding boxed area in (A). Arrows indicate BrdU positive Aqp1 expressing fibrocytes. Note that BrdU was injected in between P6 and P12, indicating that those BrdU positive Aqp1 expressing type 3 fibrocytes were long-term BrdU retaining cells. Nuclei were stained using DAPI (B). Scar bar = 100μm.

The results showed that type 3 fibrocytes in the SL have the highest capacity in the proliferation. We next asked whether they are slow-cycling cells. At P72, the BrdU immune-positive cells were observed in Aqp1 region at P72 (Fig 6A–6D). These results indicate that incorporated BrdU was retained over 8 weeks, suggesting that a part of type 3 fibrocytes in the SL may potentially be slow cycling, which is a feature of stem cells.

### 4. Repopulation of type 1 firocytes by Aqp1 expressing proliferating type 3 cells in the nose-induced damaged cochlear lateral wall

**Fig 7:**
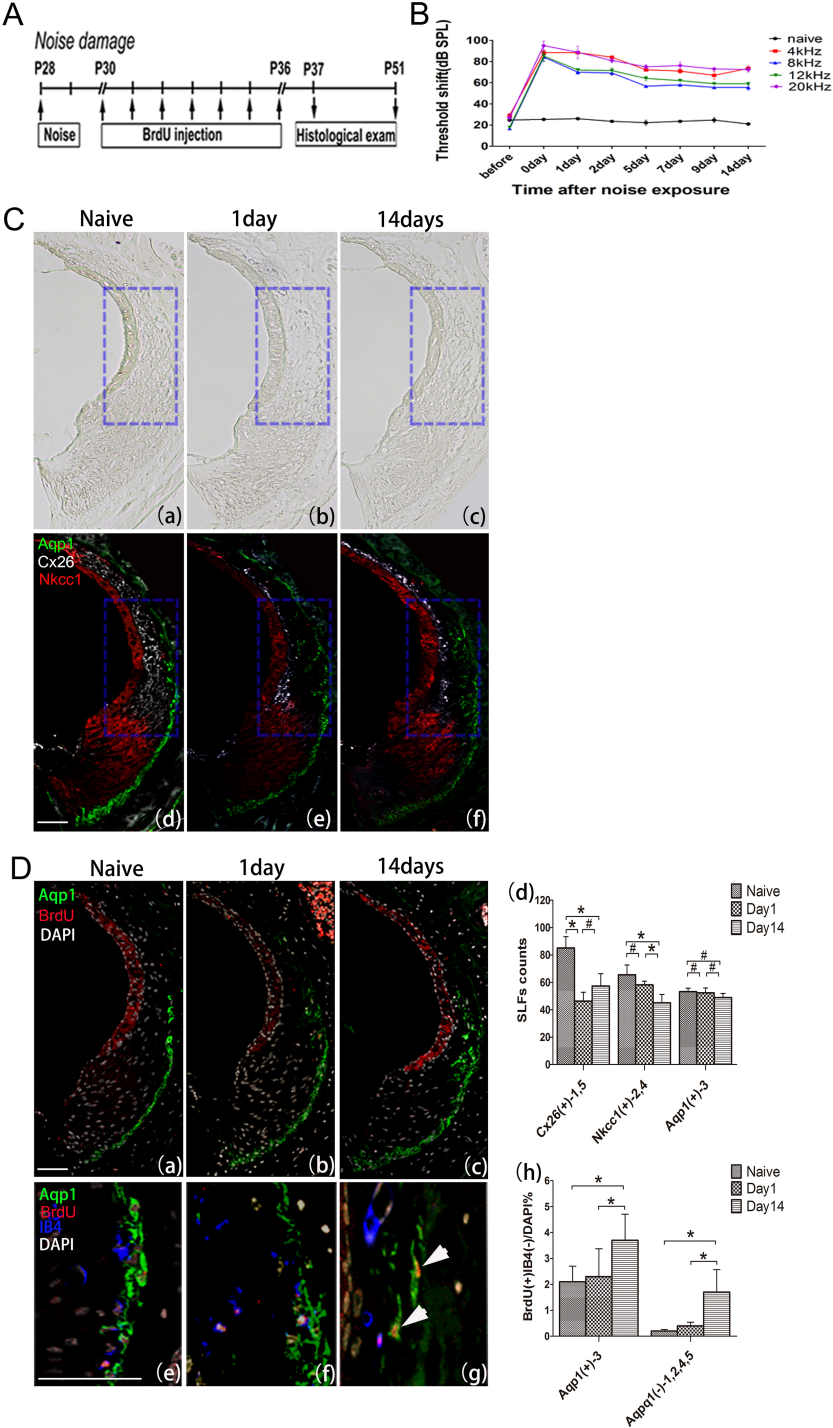
Proliferation of SLFs after intense sound exposure. Experimental design indicating the duration of BrdU treatment and observation time points in noise-damaged mice (A); Time course of threshold shifts following exposure to a 120 dB SPL of octave band noise for 2h (B); Histological evaluation (C(a-c)) and triple immunostaining for NkCC1/Cx26/Aqp1 (C(d-f)), BrdU/IB4/Aqp1 (D(e-g)) were performed at 1 day and 14 days after noise exposure. Some BrdU^+^IB4^-^ type 3 SLFs even entered type 1 area (D(g)). The number of type 3 fibrocytes did not change significantly (D(d)), but the number of BrdU incorporating type 3 fibrocytes increased during the time-course (D(h)). Arrow heads: BrdU^+^IB4^-^Aqp1^+^ fibrocytes. ^#^P > 0.05, *P < 0.05. Scar bar = 100μm.

The results above suggest that resident somatic stem cells may exist in the cochlear lateral wall and most frequently in type 3 fibrocytes. We next performed an experiment with an intense acoustic trauma model, in which lateral wall spiral ligaments are severely damaged (Fig 7A). Before noise exposure, each animal showed normal cochlear function. After noise exposure to a 120 dB SPL of octave band noise for two hours, ABRs were recorded immediately (0 day), 1, 2, 3, 5, 7, 9, 14 days after acoustic trauma, respectively, and the stable threshold shift at 4, 8, 12 and 20 kHz were confirmed at 2 weeks after noise exposure (Fig 7B). Histologically, a prominent loss of fibrocytes in type 1 area was induced with the enlarged extra cellular spaces observed at 1 day after acoustic trauma, which was repopulated after 14 days (Fig 7C). Immunohistochemistry also showed a decrease in Cx26 positive type 1 fibrocytes at 1 day after exposure (Fig 7C (d-f)), while there was no significant changes in the number of type 3 fibrocytes (Fig 7D (a-c)). Interestingly, the repopulated fibrocytes in type 1 area were positive for a type 3 marker Aqp1(Fig 7D(e-h)). When we counted the number of BrdU^+^IB4^-^ cells, the number of the fraction in type 3 SLFs area was notablely increased at 2 weeks after noise damage.

**Fig 8:**
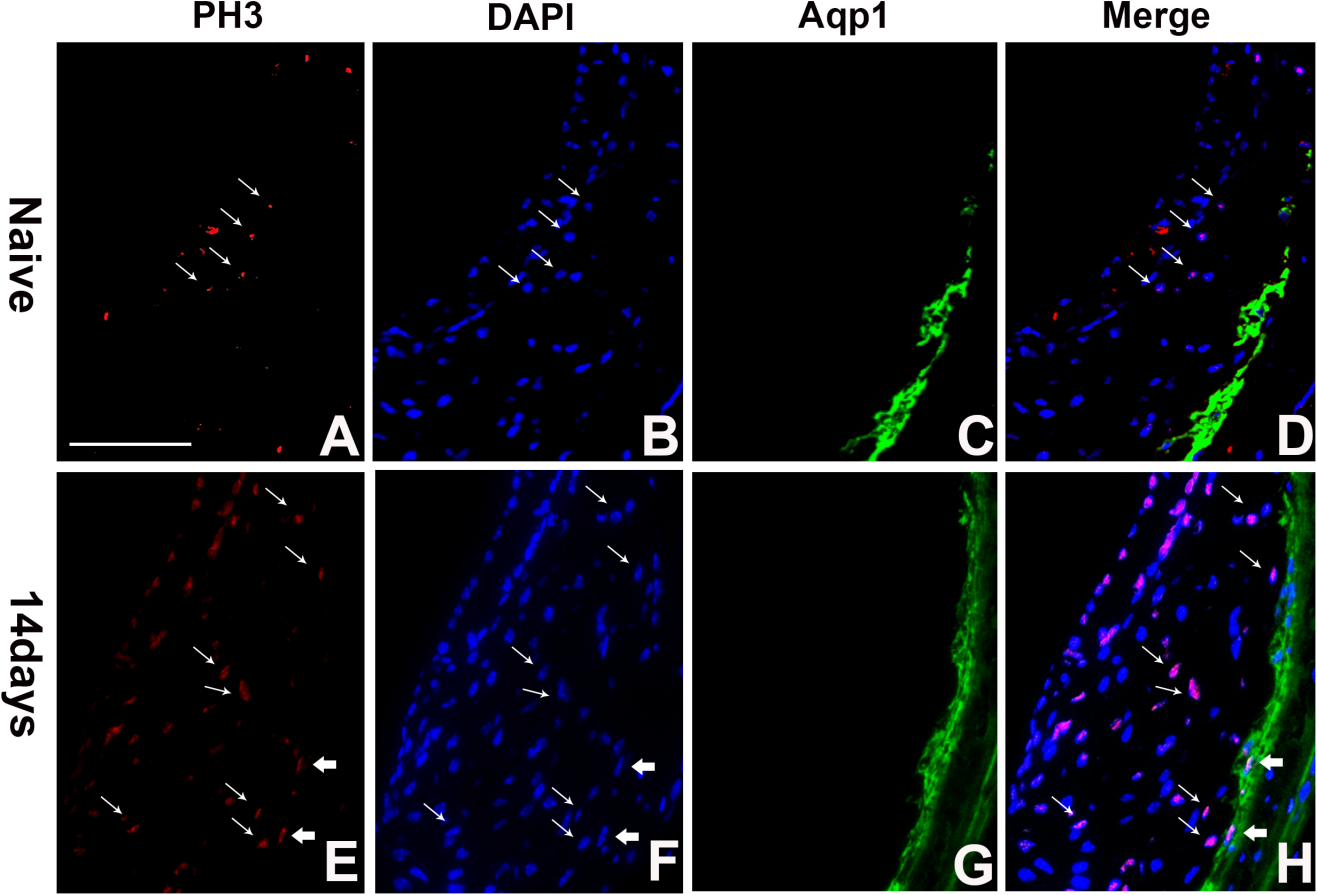
Proliferation of type 3 fibrocytes after intense sound exposure. Double immunostaining was performed for PH3 (A and E) and Aqp1 (C and G) simultaneously, and nuclei were stained using DAPI (B and F). D is a merged image of A, B and C. H is a merged image of E, F and G. We observed PH3 positive cells increased endothelial cells (thin arrows) and type 3 fibrocytes (thick arrows) after acoustic damage. Scar bar = 50μm.

To further examine the characteristics of proliferating SLFs, we performed the immunostaining for PH3, a marker for cells in G2/M phase in the cell cycle. After acoustic damage, PH3 positive cells increased significantly in type 3 fibrocytes (Fig 8), further demonstrating that some adult SLFs can enter mitosis in respond to the intense sound.

## Discussion

Hearing loss is one of the most common disabilities in the world, particularly in aged populations. In general, sensorineural hearing loss is irreversible once it has occurred because the loss of sensory hair cells is permanent in humans and other mammals. In contrast to the sensory cells, fibrocytes in the SL have been found to repopulate themselves after damage [4, 5, 10, 11].However, until now we did not clearly understand their proliferative and differentiative capacity especially during a long period after birth, which is closely related to their regenerative ability. In the present study, we used pulse-chase experiments to identify the localization and extent of label retaining cells *in vivo* with a BrdU injection while subsequent cell movement was monitored by BrdU pulse-labelling at various chase periods. We found that, compared to other cochlear regions including Organ of Corti, the spiral ganglion, tympanic border cells, the lateral wall region had significantly higher percentage of the long-term BrdU retaining cells, in fact from P13 to P72. Interestingly, most of the BrdU retaining cells in the lateral wall were located in the SL before P28 and cycled slowly because BrdU was retained as long as 72 days. Immunohistochemistry revealed that these cells were SLFs and the examination at different time points revealed that the number of those cells declined as the mice aged. Since slow-cycling cells in general contain a stem/progenitor cell population, manipulating those fibrocytes may accelerate repopulation, leading to the development of promising therapeutic strategies for treating some types of sensorineural hearing loss, such as presbyacusis [25].

In this study, some of the BrdU incorporated cells in the SL were positive for IB4, the vascular endothelial cells of a specific marker and many of the cells were negative for IB4, which were true SLFs. Negative expression of caspase3 indicated that BrdU positive cells were not dying cells or the resting cells which were too damaged to replicate. Furthermore, those fractions were also positive for Pou3f4, which reflected their immature nature. Immature SLFs that residing in the mature SL might explain how SLFs repair or replace themselves in the early stage [26].

We also performed a late-stage BrdU injection in mice, from P35 to P42 and we did not find BrdU positive cells (data not shown). Therefore, it is likely that the majority of the SLFs stays in the post-mitotic state under homeostatic condition in this period but re-enters into mitotic state when the tissue is damaged. These phenomena are commonly seen in other tissue stem cells: For instance, hematopoietic stem cells (HSCs) are a primarily quiescent population, with around 1–3% in cycle [27]. They are efficiently activated to self-renew in response to bone marrow injury or G-CSF stimulation and after re-establishment of homeostasis, activated HSCs return to dormancy [28]. Some neural stem cells stay undifferentiated and end up in specific niches, which provide the environment necessary for the quiescent life of a stem cell [29]. Note that the minor population of potential tissue stem cells cannot be detected because the tissue stem cells is in the G0 phase.

There are five different types of SLFs based on their structural features, function, and general location [30]. Type 2, 4, 5 SFLs resorb potassium ions from the surrounding perilymph and from outer sulcus cells via the Na,K-ATPase. Type 3 SLFs line the space between types 1 and 2 fibrocytes and the bony otic capsule, and are most prevalent in the most basal region of the cochlear [31, 32]. They appear not to be connected cytoplasmically to the connective tissue gap junction network [31, 32], but may form a separately coupled compartment via Connexin 43-containing channels[33]. It has been demonstrated by Mutai et al. that the overall mitotic activity of SLFs in rat decreased obviously between P7 and P10 [24]. Our study further proved that this overall mitotic declination continued from P13 to P72. Different from Mutai’s study adding two additional lines to divide SL into three sub-areas, our study was more accurate to distinguish different subtype areas and revealed that dynamic and distinct changes in BrdU^+^IB4^-^ SLFs occur in the SL by systematically monitoring incorporation of BrdU and expression of subtype SLF marker between P13 and P42. We further demonstrated that type 3 SLFs owned higher percentage of BrdU^+^IB4^-^ cells than other subtype SLFs areas before P28, suggesting that type 3 SLFs may contain stem/progenitor cells.

In noise injured mice with the peak of 120 dB, the damage is permeant and type 4 fibrocytes are especially most vulnerable, lacking any sign of regeneration while degeneration of type 3 was never detectable by previous histopathological observations [31]. In this study, we found degeneration of type 1 SLFs that were repopulated by type 3 SLFs. In type 1 SLFs, we did not observe BrdU incorporating cells after noise damage in adult, indicating that the repopulation of type 1 SLFs was not due to their symmetric mitosis. This observation is consistent with the previous study by Mutai et.al. showing that mitotic activity of SLFs in the type 1 area is found at low level during the postnatal period and decreases as the animal ages [24]. We further proved that, after noise exposure, the number of BrdU^+^Aqp1^+^ SLFs were increased and, curiously, observed in the type1 SFL-area. One possibility is that the rapidly proliferating type 3 fibrocytes simply replace the type 1 area where cells devoid. Alternately, type3 SLFs have the migration ability of multipotent stem cells and replace type 1 SLFs most likely through trans-differentiation. Further study for the lineage tracing experiments, typically by using transgenic mice, would elucidate the issue for the future.

There are some inevitable limitations in this study. First, our estimates of BrdU incorporating cells maybe lower than the actual number because BrdU could be metabolized or degraded over time similar to other nuclear components. Second, this *in vivo* study can only assume the presence of putative stem cells, further research is expected to demonstrate these cells’ migration, multi-potency, and functional roles both *in vitro* and *in vivo* assays.

## Conclusions

We evaluated the locations and characteristics of BrdU incorporating cells as a step to determine tissue-specific stem cells in the SL. Higher percentage of BrdU^+^IB4^-^ cells were located in the type 3 area and kept in quiescent state. The acoustic injury model further implies type 3 fibrocytes can re-enter the cell cycle and begin proliferation again, and may provide surport to decreased type 1 fibrocytes. Collectively, we conclude that the type 3 cells of the spiral ligament fibrocytes have the putative tissue stem cell capacity in the cochlear lateral wall and that they may be employed for potential regenerative therapies against hearing impairment.
